# Predominance of the Rare EGFR Mutation p.L861Q in Tunisian Patients with Non-Small Cell Lung Carcinoma

**DOI:** 10.3390/genes13081499

**Published:** 2022-08-22

**Authors:** Rania Abdelmaksoud-Dammak, Nihel Ammous-Boukhris, Amèna Saadallah-Kallel, Slim Charfi, Souhir Khemiri, Rim Khemakhem, Nesrin Kallel, Wala Ben Kridis-Rejeb, Tahya Sallemi-Boudawara, Afef Khanfir, Ilhem Yangui, Jamel Daoud, Raja Mokdad-Gargouri

**Affiliations:** 1 Center of Biotechnology of Sfax, Laboratory of Eucaryotes Molecular Biotechnology, University of Sfax BP K 1177, Sfax 3018, Tunisia; 2 Department of Anatomopathology, Habib Bourguiba Hospital, Sfax 3002, Tunisia; 3 Department of Medical Oncology, Habib Bourguiba Hospital, Sfax 3002, Tunisia; 4 Department of Pneumology, Hedi Chaker Hospital, Sfax 3089, Tunisia; 5 Department of Radiotherapy, Habib Bourguiba Hospital, Sfax 3002, Tunisia

**Keywords:** non-small-cell lung carcinoma, epidermal growth factor receptor, mutation, pyrosequencing, tyrosine kinase inhibitors, targeted therapy

## Abstract

Objectives: Several new cancer therapies targeting signaling pathways involved in the growth and progression of cancer cells were developed as personalized medicine. Our study aimed to identify epidermal growth factor receptor (*EGFR*) mutations for TKI treatment in non-small-cell lung cancer (NSCLC) Tunisian patients. Methods: Analysis of the TKI sensitivity mutations in exons 18 to 21 of the *EGFR* gene and exon 15 of the *B-raf* gene was performed in 79 formalin fixed-paraffin embedded (FFPE) NSCLC samples using pyrosequencing. Results: *EGFR* mutations were detected in 34 cases among 79 (43%), with the predominance of the L861Q in exon 21 found in 35.3% of the cases (12 out of 34). Deletions in exon 19 were found in 8 cases (23.5%), and only one young male patient had the T790M mutation. Three patients harbored composite *EGFR* mutations (p.E746_A750del/p.L861R, p.E746_S752>V/p.S768I, and p.G719A/p.L861Q). Furthermore, the *EGFR* mutated status was significantly more frequent in female patients (*p* = 0.019), in non-smoker patients (*p* = 0.008), and in patients with metastasis (*p* = 0.044). Moreover, the *B-raf* V600E was identified in 5 *EGFR* negative patients among 39 analyzed samples (13.15%). Conclusion: The p.L861Q localized in exon 21 of the *EGFR* gene was the most common mutation identified in our patients (35.3%), whereas the “classic” *EGFR* mutations such as Del19 and p.L858R were found in 23.5% and 11.7% of the cases, respectively. Interestingly, most of p.L861X mutation-carrying patients showed good response to TKI treatment. Altogether, our findings suggest a particular distribution of the *EGFR*-TKIs sensitivity mutations in Tunisian NSCLC patients.

## 1. Introduction

Lung cancer is the most common cause of cancer deaths per year, estimated to be responsible for nearly one in five deaths worldwide, with about 2.2 million cases in 2018 [1]. According to 2020 GLOBOCAN data, lung cancer was the second most commonly diagnosed cancer (11.4%) with 1.8 million evaluated deaths (18%) [1]. In Tunisia, lung cancer is the 2nd most common cancer with around 2929 new cases per year and a frequency of 15.1% [2]. This malignancy is the most lethal cancer with a mortality rate of 22.2% in 2020 [3].

Non-small cell lung cancer (NSCLC) is the major type of lung cancer, identified in 80 to 85% of patients, for which adenocarcinoma is the most common subtype [4]. More than 60% of lung cancer diagnoses are made at an advanced stage (III or IV) and the 5-year overall survival rate for metastatic NSCLC remains very poor [4].

In NSCLC, numerous molecular alterations affecting key signaling pathways have been reported and defined as driver oncogenes. Epidermal growth factor receptor (*EGFR*) is a transmembrane glycoprotein encoded by the *ErbB* gene and overexpressed in more than 40% of non-small cell lung cancers (NSCLC) [5,6,7,8,9]. *EGFR* is mutated in 10–20% of lung adenocarcinomas among Caucasian patients and more commonly in young never-smoker Asian women, whereas *EGFR* mutations are rare in other lung cancer subtypes [10,11].

Several drugs have been developed for *EGFR*-driven NSCLC and are becoming the standard of care for the first-line treatment of advanced NSCLC with *EGFR* mutations [12]. Several somatic mutations in the *EGFR* gene have been identified and classified as conferring sensitivity to the *EGFR* tyrosine kinase inhibitors (TKIs), which are mainly located in the intracellular tyrosine kinase domain spanning from exon 18 to exon 21 [12]. The “classic” *EGFR* mutations, namely deletions in exon 19 and L858R in exon 21, account for about 85% of *EGFR* mutations in NSCLC [13,14,15]. Patients harboring either of these two mutations represent a classic subtype of NSCLC, with a higher response to *EGFR*-TKIs and even improved overall survival (OS) rates compared to patients with wild-type *EGFR* tumors [16,17,18]. On the other hand, rare mutations including point mutations, deletions, and insertions within exons 18 to 21 of the *EGFR* gene account for the remaining 15% of the *EGFR* mutations in NSCLC [13,14,15].

*B-raf* is one of three members of the RAF kinase family: *A-raf*, *B-raf*, and *C-raf*, which belongs to the group of serine-threonine kinases and plays a critical role in mitogen-activated protein kinase (MAPK) pathways [19,20]. Mutations in *B-raf*, mainly the V600E, have been found in different types of cancer, predominantly melanoma and metastatic colorectal cancer with frequencies of 50% and 9%, respectively [21,22]. In NSCLCs, the *B-raf* V600E was reported in 1 to 3% of the cases and generated a constitutive activation of the MAP pathway, leading to cell growth, proliferation, and resistance to negative modulatory feedback signals [23].

Several inhibitors of the *B-raf* V600E mutant protein, such as dabrafenib, are recently used in therapy and have shown increased response in patients carrying the V600E mutation, with an average of 5.5 months progression-free survival [24,25].

In Tunisian NSCLC patients, only few studies have investigated the *EGFR* and *B-raf* mutations. Using different methods such as immunohistochemistry, QPCR, or NGS, the percentage of *EGFR* mutations varied from 18.4 to 44% [26,27,28,29]. The most frequent mutations were exon 19 deletions and the p.L858R in exon 21 [27,28,29].

Regarding *B-raf* mutation, the study of Mezni et al. reported that among 41 patients screened by NGS, only 3 harbored mutations [28].

In order to better investigate the profile of *EGFR* and *B-raf* in NSCLC the present study aims to identify the EGFR and *B-raf* mutations in 79 Tunisian patients. *EGFR* mutations were correlated with clinicopathological features, treatment, and patient’s survival.

## 2. Materials and Methods

### 2.1. Clinical Samples

Between 1 March 2018 and 30 June 2022, 79 tumor samples were collected from Tunisian patients with an advanced lung adenocarcinoma. Clinicopathological features were available for only 69 cases. The average age was 59.54 years (range 24–85 years). In our cohort, 69.56% (48/69) of patients had metastases and 61.21% (44/69) declared a previous or current smoking history. Available characteristics of patients included in this study are shown in Table 1.

All analyses were conducted with respect of patients’ confidentiality and according to procedures approved by the Personal Protection Committee (PPC) of UHC Habib Bourguiba of Sfax, Tunisia, responsible for ethics in research. All samples were histologically analyzed by a pathologist, the percent of tumor cells was determined, and each sample was classified according to the WHO classification of lung cancer.

### 2.2. DNA Extraction and PCR Amplification

Formalin-fixed and paraffin-embedded tissues genomic DNA was extracted from 3–6 (10 μm thick) sequential sections through QIAamp DNA FFPE tissue kit (Qiagen) according to the manufacturer’s instructions and checked for adequacy by NanoDrop. Primers used were previously developed in our lab and each sample was analyzed using both our primers and the Therascreen *EGFR* Pyro Kit primers (Qiagen) for validation. In brief, when using our own developed primers, PCR reactions were performed on 100 ng of DNA in a total volume of 50 μL containing 5× buffer, 200 μM dNTP, 0.5 μM of each primer, and 0.2U Taq polymerase (Takara), with the following cycling conditions: 95 °C for 5 min followed by 40 cycles of denaturation at 95 °C for 30 s, annealing at 56/60 °C for 30 s, extension at 72 °C for 30 s. A final extension at 72 °C for 5 min was finally performed. PCR of the Therascreen *EGFR* Pyro Kit were performed according to the manufacturer directives (Qiagen). Amplicons were analyzed by gel electrophoresis on a 2% agarose gel stained with ethidium bromide and visualized by ultraviolet trans-illumination. 

### 2.3. Pyrosequencing Analysis

PCR products were incubated under shaking with binding buffer (40 µL) and added with sepharose beads (1 μL) covered by streptavidin. Then, PCR products were washed with 70% ethanol, denatured with denaturation solution (Qiagen), and re-washed with wash solution (Qiagen). A pyrosequencing reaction was then performed for AQ mode in a total of 25 μL, including 24.2 μL of annealing buffer and 0.8 μL of sequencing primer (final concentration 0.3 μM). Pyrosequencing assays were performed on a PyroMark Q24 MDx using PyroMark Gold reagents (Qiagen). Assays for mutation analysis in exons 18, 19, 20, and 21 of *EGFR* and exon 15 of *B-raf* were created according to manufacturer’s instructions and nucleotide dispensation order was outlined by the software Q24 2.0. Sequencing primers were generated according to PyroMark Assay Design software version 2.0 (Qiagen). Pyromark Q24 ID version 2.0.8 software was used to generate and automatically analyze pyrograms resulting from sequencing onto PyroMark Q24 ID system.

### 2.4. Statistical Analysis

The SPSS software Version 20 was used to statistically analyze the association between *EGFR* and *B-raf* mutation state and clinicopathological features, and the correlations were assessed by Chi-square test. Statistical significance was set to *p* ≤ 0.05 in each test.

## 3. Results

### 3.1. EGFR and BRAF Mutation Analysis

**Figure 1:**
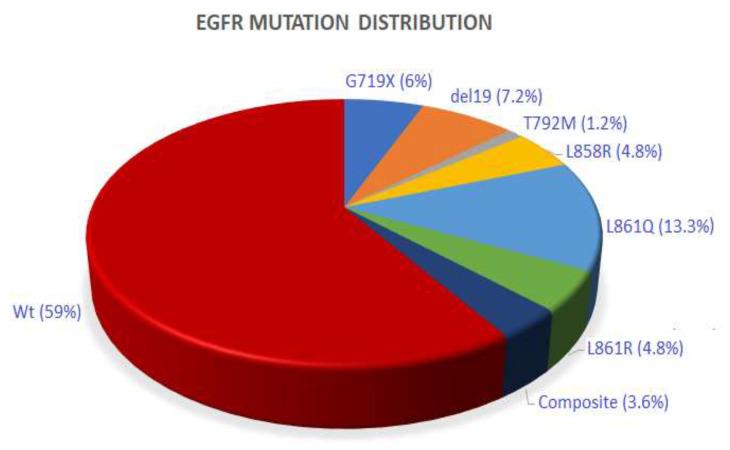
Histogram showing distribution of identified *EGFR* mutations.

Mutation screening of exons 18 to 21 of the *EGFR* gene showed that 34 cases among 79 (43%) were mutated. Among *EGFR*-positive patients, 35.3% (12 out of 34) carried the p.L861Q mutation, while the p.L861R was identified in only 5 patients (Figure 1, Table 2). Regarding the “classic” mutations, deletions in exon 19 were detected in 23.5% (8/34) of cases with the predominance of the p.E746_A750del (5 among 8 cases, 62.5%), while 11.7% (4 out of 34) carried the p.L858R (Figure 1, Table 2). In addition, composite mutations were identified in 3 patients: p.E746_A750del/p.L861R, p.E746_S752>V/p.S768I, and p.G719A/p.L861Q (Table 2).

Furthermore, 38 *EGFR* negative patients were screened for the V600E mutation in the *B-raf* gene by pyrosequencing. Only 5 patients (13.15%) carried the V600E *B-raf* mutation.

### 3.2. EGFR Mutations and Clinicopathological Features

**Figure 2:**
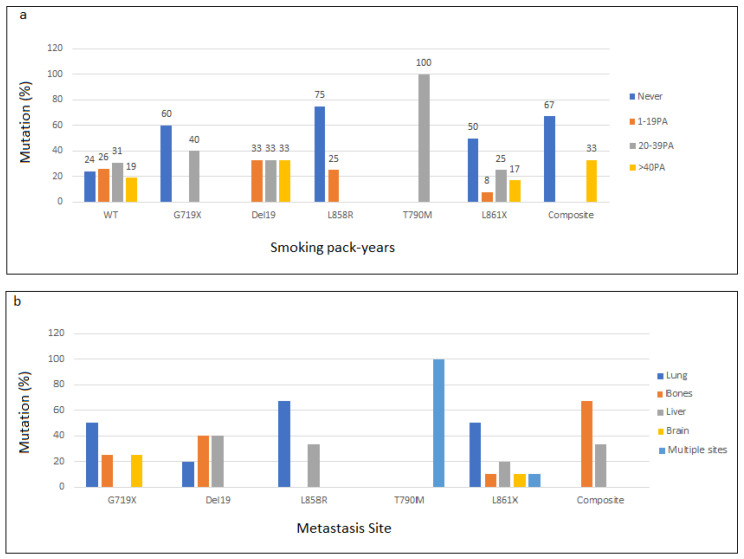
Distribution of EGFR mutation according to smoking status (**a**) and metastasis site (**b**).

*EGFR* mutation correlated significantly with gender (*p* = 0.019), non-smoking history (*p* = 0.008), and metastasis (*p* = 0.044) (Table 3). In our cohort, 44 patients are current orformer smokers and 18 among them carried *EGFR* mutation. Figure 2a represent mutation distribution according to the number of smoked pack year (PA).

Further, we noticed that about 50% (10 among 22) of patients with distant metastasis harbored the p.L861Q or p.L861R in *EGFR* exon 21. All 3 patients with composite mutations had bone or hepatic distant metastasis, as presented in Figure 2b. 

### 3.3. EGFR and BRAF Mutations and Therapy

Regarding anti-cancer treatment, only a few patients carrying *EGFR* mutations had received TKI-based therapy (Table 4). Indeed, out of the 12 *EGFR* positive patients, for whom we had access to therapy and follow-up data, only 6 were able to benefit from erlotinib targeted therapy, and most of them (5 patients) harbor the p.L861X, alone or associated to exon 19 deletion.

The first case is a 65-year-old woman (P27), initially diagnosed with a localized form of NSCLC and treated by conventional chemotherapy and radiotherapy. After developing metastasis, she tested positive for the p.L861Q mutation and had received a 1-year long erlotinib therapy. Now she is in complete remission with an OS of 40 months (Table 2)

On the other hand, the 50-year-old patient (P36) had NSCLC with bone metastasis and carried composite *EGFR* mutations, namely p.E746_A750del/p.L861R, had received 2 months of erlotinib therapy, during which he showed a 63% tumor regression. During the third month of treatment, he displayed a resumption of tumor progression, typical of the appearance of resistant mutations. Having been unable to find those mutations in the circulating tumor DNA, he is currently undergoing chemotherapy while waiting to be re-biopsied and re-tested for these mutations.

Patient P140, who also had NSCLC with bone metastasis, had been treated with several rounds of chemotherapy before being tested for *EGFR* mutations. After discovering the p.L861Q mutation, he was treated with erlotinib for only 2 weeks, after which he died of interstitial lung disease.

Patients P149, P157, and P160 are still alive and have been on erlotinib therapy for 1 month or 2, and we do not have enough hindsight to judge the effectiveness of erlotinib therapy in their case.

As for patients carrying the *B-raf* V600E mutation, follow-up and treatment data were available for one patient only. This patient, who is a 70-year-old man, had NSCLC with hepatic, adrenal, and cerebral metastasis. He was treated with chemotherapy for 5 months before being tested for *EGFR* and *B-raf* mutations. Despite having the V600E mutation, he did not have the chance to benefit from targeted therapy, since he passed away 7 months after being initially diagnosed.

## 4. Discussion

In NSCLC, the incidence of *EGFR* mutations varies considerably in different regions of the world. Several studies reported that the prevalence of *EGFR* mutations ranged from 11% to 50% [30,31]. A large meta-analysis conducted in 2016 including 456 studies showed significant heterogeneity in all analyzed variables related to the prevalence of *EGFR* mutations in NSCLC patients [32]. In fact, ethnic backgrounds, patient characteristics, clinical settings, and methodology may contribute to these differences. This was confirmed by numerous reports from frequently assessed populations, showing a huge variability of *EGFR* mutation frequencies in patients with lung adenocarcinoma, ranging between 6 and 41% in Europe, 3 and 42% in North America, and 20 and 76% in Asia-Pacific [33].

Our studied population, which is predominantly from South Tunisia, had a percentage of 43% *EGFR* positivity. This rate is comparable to those observed in Asian countries and clearly higher than in Europe and North America. A meta-analysis including Middle Eastern and African studies reported a prevalence of 21.2% of *EGFR* mutations, varying from 44% in Turkey to 21% in Morocco and 2.1% in Saudi Arabia [34]. In addition, two recent studies from Morocco and Algeria revealed *EGFR* mutation frequencies of 21.9%, and 39.6%, respectively [35,36]. Analysis of the *EGFR* TKI sensitivity mutations in Tunisian NSCLC patients showed variable frequencies, depending on the number of analyzed samples, the patient’s selection criteria (clinical and pathological), and the used mutational analysis technique. In fact, while Mraihi et al. found 44% *EGFR* positive samples using IHC, Dhieb et al. reported only one sample with the E746-A750 del19 mutation, using the same technique [26,27]. Another study evaluating the molecular profile of 87 NSCLC samples by qPCR or NGS found that 18.4% of patients had *EGFR* activating mutations (12 cases with the exon 19 deletions and 4 patients carrying the p.L858R) [28]. In addition, Arfaoui et al. showed that 3 out of the 26 analyzed samples harbor two sensitizing mutations (exon 19 deletion and p.G719X) and one exon 20 insertion associated with de novo resistance to targeted *EGFR* inhibitors and correlate with a poor patient prognosis [29]. Altogether, these findings support the heterogeneity in the prevalence of *EGFR* mutations among populations.

Interestingly and apart from the variability in the *EGFR* mutation frequencies, our results showed a particularity in the mutation profile of NSCLC patients. Actually, in-frame deletions of amino acids LREA of exon 19 and the p.L858R mutation are considered the “classic” mutations, accounting for 85% of *EGFR* mutations [11,12]. In the present study, we found a higher frequency of the p.L861X mutation with 35.3% and 14.7% of cases carrying the p.L861Q and the p.L861R, respectively. In a recent study, John et al. reported that the prevalence of uncommon *EGFR* mutations varied between 1.0% and 18.2% in Asia and South America [37]. According to this study, the most frequently reported uncommon mutations were G719X (0.9–4.8%), exon 20 insertions (0.8–4.2%), L861X (0.5–3.5%), and S768I (0.5–2.5%). Compared to our results, the p.L861X was more frequent in NSCLC Tunisian patients (50%) with the predominance of the p.L861Q mutation. Interestingly, among the p.L861X patients, 5 out of 7 received TKI-based therapy and showed a good response compared to those who were treated with only chemotherapy. In this context, Chiu et al. concluded that the p.L861Q is somewhat sensitive to TKIs but to a lesser extent than the “classic” mutations [38]. In addition, Liu et al. investigated the sensitivity to six first-in-class TKIs of the rare p.L861Q mutation by establishing two cell lines (*EGFR* p.L861Q variant and *EGFR* p.L861Q + exon 19 deletion variant) using the CRISPR-Cas9 gene-editing technology [39]. The authors showed that the *EGFR* p.L861Q + 19del variant and p.L861Q variant displayed significant sensitivity to TKIs tested particularly to gefitinib [39].

Moreover, a meta-analysis reported that afatinib had good clinical activity in NSCLC with uncommon p.L861Q mutation, with a response rate of 56.3%, a median progression-free survival of 8.2 months, and a median overall survival of 17.1 months [40].

Another recent case report showed the successful treatment of an 83-year-old patient with an uncommon L861Q epidermal growth factor receptor mutation. He was treated with low-dose afatinib, supporting the sensitivity of this mutation to TKI-based therapy [41].

Regarding the “classic” mutations such as deletion in exon 19 and p.L858R, we found that 23.5% of our patients carried deletion in exon 19, which is in line with data from previous studies including those conducted on Tunisian patients [26,27,28,29,30,31,32,33,34,35,36]. However, the p.L858R was identified in only four patients (11.7%), confirming once again the particularity of our cohort.

In addition, three patients harbored composite *EGFR* mutations, two cases carried an exon 19 deletion associated with p.L861R or p.S768I, and the third patient harbored the p.G719A with the p.L861Q. Composite *EGFR* mutations are double or multiple mutations of the *EGFR* tyrosine kinase domain, in which a sensitizing mutation is identified along with another one, usually of unclarified clinical significance [42]. Double mutations are detected in 14 to 18% of NSCLC samples, but their clinical significance remains not clearly characterized [43,44]. Kim et al. concluded that patients with composite *EGFR* mutations have poor clinical outcomes and should be closely monitored during follow-up [41]. In our study, the follow-up was available for only one patient among three carrying composite *EGFR* mutations. This 50-year male patient carrying the p.E746_A750del/p.L861R was treated with erlotinib and had a survival rate of 15 months, but he developed resistance to the TKI, suggesting the emergence of resistance mutation. Recently, Liu et al. reported a case of a female patient with lung adenocarcinoma carrying three mutations in *EGFR* exon 18: p.G724S, p.E709K, and p.V689I. The patient developed resistance to multiple *EGFR*-TKI and had a short overall survival time [45].

In addition to *EGFR*, the *B-raf* gene alterations are also associated with increased kinase activity leading to constitutive activation of the MAP kinase pathway [23]. *B-raf* mutations have been reported in about 4% of NSCLC cases and are commonly associated with adenocarcinoma non-small cell lung cancer [46]. *B-raf* V600E mutation specifically occurs in about 1–2% of non-small cell lung cancer patients and most patients harboring this genetic alteration tend to have a smoking history [47]. Our findings are higher than the global data with a mutation frequency reaching 13.5%. In Tunisian patients, Mezni et al. reported only 2 cases harboring the V600E mutation in a cohort of 41 patients [28]. Two large meta-analyses concluded that there was a significant association between *B-raf* mutations and adenocarcinomas in NSCLC compared with non-ADKs and no significant difference was observed in smoking and stage in patients with *B-raf* mutations [48,49].

Finally, this study has the convenience of being the first Tunisian study enrolling 79 patients that are locally analyzed by pyrosequencing. However, it still has some limitations, such as the limited cohort size, the lack of patient follow-up, and the used therapy protocol. Further studies with larger samples and clinical data are required.

## 5. Conclusions

In conclusion, we found that the p.L858R usually defined as the “classic” *EGFR* mutation is rare in Tunisian patients in contrast with previous reports. Inversely, the p.L861Q is predominant and identified in 35% of cases. Interestingly, the p.L861X patients showed a good response to erlotinib compared to those who were treated with only chemotherapy. Those findings are of a great importance for clinicians to better manage Tunisian NSCLC patients. However, a study on a much bigger cohort is needed to validate these results. 

## Figures and Tables

**Table 1:** Clinical pathological characteristics of patients.

Patients’ Characteristics	Number (%)
**Total**	**79**
**Age (Years)**	**69**
Median	59.54
Range	24–85
**Gender**	**79**
Male	65 (82.27)
Female	14 (17.72)
**Smoking history**	**69**
Never-smoker	24 (34.78)
Former/current smoker	44 (61.21)
**Histological Type**	**56**
Adenocarcinoma	51 (91.07)
epidermoid carcinoma	4 (7.14)
Pleomorphic carcinoma	1(1.78)
**Metastasis**	**69**
Absence	48 (69.56)
Presence	21 (30.43)

**Table 2:** Identified mutation in exons 18–21 of the *EGFR* in Tunisian NSCLC patients. *: Deceased before starting the target therapy.

Patient	Gender	Age	*EGFR* Alteration	Metastasis	Smoking History	Therapy	Status	Survival (Months)
P3	M	60	p.L861Q	Lymph nodes	30 PA	Chemotherapy	dead	37
P4	M	46	p.T790M	Liver, Bones	20 PA	-	-	-
P27	F	65	p.L861Q	Lung	No	Chemotherapy + Erlotinib	alive	40
P34	M	72	p.L861R	No	25 PA	Chemotherapy	dead	18
P35	M	-	p.L861R	No	30 PA	-	-	-
P36	M	50	p.E746_A750del/p.L861R	Bones	40 PA	Erlotinib	alive	15
P37	M	60	p.G719S	Brain	No	Chemotherapy	dead	35
P40	M	72	p.G719A	Lung	No	-	-	-
P42	M	63	p.E746_A750del	Bones	60 PA	Chemotherapy	alive	17
P104	M	-	p.L861Q	-	20 PA	-	-	-
P105	M	-	p.E746_A750del	Liver	40 PA	-	-	-
P106	F	-	p.E746_S752>V/p.S768I	Bones	No	-	-	-
P109	F	-	p.E746_T751>I	Bones	10 PA	-	-	-
P112	M	-	p.L861R	Brain	50 PA	-	-	-
P117	F	-	p.L747_T751del	Bones	15 PA	-	-	-
P120	M	66	p.G719A/p. L861Q	Liver	No	-	-	-
P121	M	51	p.L858R	Lung	No	-	-	-
P125	M	54	p.E746_A750del	Lung	30 PA	-	-	-
P128	M		p.L858R	Liver	No	-	-	-
P134	M	67	p.L861Q	No	No	-	-	-
P135	M	54	p.G719C	No	40 PA	-	-	-
P136	M	76	p.E746_A750del	No	20 PA	Surgery	alive	7
P137	F	63	p. L861Q	No	No		-	-
P139	M	64	p. G719C	Adrenal	40 PA	Chemotherapy	alive	18
P140	M	66	p. L861Q	Bones	Unknown	Chemotherapy + Erlotinib	dead	1
P145	M	59	p.L858R	No	50 PA	*	dead	3
P146	F	48	p.G719A	Bones	No	*	dead	4
P148	F	70	p. L861Q	No	No	-	-	-
P149	F	60	p. L861R	No	No	Erlotinib	alive	3
P150	M	62	p. L861Q	No	No	-	-	-
P156	M	62	p. L861Q	Liver	40 PA	-	-	-
P157	M	54	p. L861Q	Liver	No	Erlotinib	alive	2
P158	M	56	p. L861Q	Liver	30 PA	-	-	-
P160	F	75	p. L858R	Bones	No	Erlotinib	alive	2

**Table 3:** *EGFR* mutations and clinicopathological feature of Tunisian NSCLC.

Clinical Feature		Overall	Mutation (%)	Wt (%)	*p*-Value
**Gender**	M	65 (82.27)	24 (36.9)	41 (63.1)	**0.019**
	F	14 (17.72)	10 (71.4)	4 (28.6)	
**Age**	<60	26 (32.91)	10 (38.5)	16 (61.5)	0.307
	>60	33 (41.7)	16 (48.5)	17 (51.5)	
	Unknown	20 (25.31)	8 (40)	12 (60)	
**Smoking History**	Smoker	45 (56.6)	15 (33.3)	30 (66.7)	**0.008**
	Non-smoker	24 (31.3)	16 (61.5)	10 (38.5)	
	Unknown	10 (12)	3 (30%)	7 (70%)	
**Histological subtype**	Adenocarcinoma	51 (64.55)	21 (41.2)	30 (58.8)	0.646
	Other subtypes	5 (6.32)	4 (80)	1 (20)	
	Unknown	23 (29.11)	9 (39.1)	14 (60.9)	
**Metastasis**	Presence	48 (60.75)	26 (54.2)	22 (45.8)	**0.044**
	Absence	21 (26.58)	6 (28.6)	15 (71.4)	
	Unknown	10 (12)	2 (20)	8 (80)	

**Table 4:** Treatment response and survival of patients carrying *EGFR* mutations.

Patient	*EGFR* Alteration	Therapy	Response to Therapy Protocol	Status	Survival (Months)
**P3**	p.L861Q	Chemotherapy	Good response to chemotherapy then metastasis	died	37
**P27**	p.L861Q	Chemotherapy + Erlotinib	Complete remission	alive	40
**P34**	p.L861R	Chemotherapy	Bad response to chemotherapy	died	18
**P36**	p.E746_A750del/p.L861R	Erlotinib	63% tumor regression then resumption of tumor progression	alive	15
**P37**	p.G719S	Chemotherapy	Bad response to chemotherapy	died	35
**P42**	p.E746_A750del	Chemotherapy	Good response to chemotherapy	alive	17
**P136**	p.E746_A750del	Surgery	No treatment	alive	7
**P139**	p. G719C	Chemotherapy	Good response to chemotherapy	alive	18
**P140**	p. L861Q	Chemotherapy + Erlotinib	Chemotherapy then 2 weeks of erlotinib, died of interstitial lung disease	died	1
**P149**	p. L861R	Erlotinib	Treated for 1 month	alive	3
**P157**	p. L861Q	Erlotinib	Treated for 2 months	alive	2
**P160**	p. L858R	Erlotinib	Treated for 1 month and 2 weeks	alive	2

## Data Availability

The data presented in this study are available on request from the corresponding author. The data are not publicly available due to patient’s information privacy.

## List of Abbreviations

EGFR	Epithelial Growth Factor Receptor
TKI	Tyrosine Kinase Inhibitors
NSCLC	Non-Small-Cell Lung Carcinoma
FFPE	Formalin-Fixed Paraffin-Embedded
ErbB2	Erb-B2 Receptor Tyrosine Kinase 2
MAPK	Mitogen-Activated Protein Kinases
WHO	World Health Organization
PA	pack years
IHC	Immunohistochemistry
Wt	Wild type
